# Alternatives to Transplantation in the Treatment of Heart Failure: New Diagnostic and Therapeutic Insights

**DOI:** 10.1155/2015/278163

**Published:** 2015-11-29

**Authors:** Francesco Nicolini, Massimo F. Piepoli, Giulio Agnetti, Giuseppe Siniscalchi

**Affiliations:** ^1^Cardiac Surgery Unit, Department of Clinical and Experimental Medicine, University of Parma, Via A. Gramsci 14, 43126 Parma, Italy; ^2^Heart Failure Unit, Cardiology Department, Guglielmo da Saliceto Hospital, 29121 Piacenza, Italy; ^3^Division of Cardiology, Johns Hopkins University School of Medicine, Baltimore, MD 21205, USA; ^4^DIBINEM, University of Bologna, 40126 Bologna, Italy; ^5^Department of Cardiovascular Surgery, University Hospital Lausanne, 1011 Lausanne, Switzerland

The aim of our current special issue was to present a series of original researches and reviews on recent advances in the diagnosis, medical therapy, and surgical approaches of heart failure.

As reported in the introductive review of Agnetti et al., cardiovascular disease is the leading cause of mortality in the US and in westernized countries with ischemic heart disease accounting for the majority of these deaths. Paradoxically, the improvements in the medical and surgical treatments of acute coronary syndromes are leading to an increasing number of “survivors” who are then developing heart failure. Despite considerable advances in its management, the gold standard for the treatment of end-stage heart failure patients remains heart transplantation. Nevertheless, this procedure can be offered only to a small percentage of patients who could benefit from a new heart due to the limited availability of donor organs. The authors reported in this comprehensive review the evaluation of the safety and efficacy of innovative approaches in the diagnosis and treatment of patients refractory to standard medical therapy and excluded from cardiac transplantation lists.

Among the studies included in this special issue, two of them investigated specific pathogenic aspects of heart failure. M. Kunin et al. studied the role of proinflammatory cytokines in congestive heart failure. In particular the authors evaluated the effect of peritoneal dialysis used in the long-term management of these patients on the peripheral-circulating levels of these cytokines. Interestingly, they found that peritoneal dialysis treatment caused a reduction in circulating inflammatory cytokines levels along with improvement in plasma markers of inflammation in patients with refractory chronic heart failure, concluding that this effect may be partly responsible for the efficacy of peritoneal dialysis for refractory heart failure.

It is described that heart failure is accompanied by the development of an imbalance between oxygen- and nitric oxide-derived free radical production leading to protein nitration. To cast further light on this issue, A. Cabassi et al. investigated the relationship between plasma myeloperoxidase-related chlorinating activity, ceruloplasmin, and ferroxidase I and nitrosative stress and inflammatory, neurohormonal, and nutritional biomarkers in heart failure patients. This elegant study supported the conclusions that plasma myeloperoxidase chlorinated activity is increased in elderly patients who suffer from chronic heart failure and positively associated with ceruloplasmin and inflammatory, neurohormonal, and nitrosative parameters, suggesting a key role in heart failure progression.

A major focus of studies on acute heart failure is the need for methods that allow the early detection of hemodynamic variables that can be a key prognostic role after cardiac surgery. F. Corradi et al. in their study investigated the Renal Doppler Resistive Index as a marker of oxygen supply and demand mismatch in 61 postoperative cardiac surgery patients. Interestingly, by multivariate analysis, Renal Doppler Resistive Index was significantly correlated with mixed-venous oxygen saturation, suggesting that, in mechanically ventilated patients after cardiac surgery, it could be used as a marker of early vascular response to tissue hypoxia.

We must not forget that there are many patients suffering from heart failure secondary to extracardiac diseases. Among the causes of heart failure, an important place is held by cardiotoxicity due to antineoplastic treatments that has emerged as a clinically relevant problem as a consequence of the relevant improvement of survival after cancer. In the last decade recent advances have emerged in clinical and pathophysiological aspects of left ventricular dysfunction induced by the most widely used anticancer drugs. M. Molinaro et al., in their comprehensive review entitled “Recent Advances on Pathophysiology, Diagnostic and Therapeutic Insights in Cardiac Dysfunction Induced by Antineoplastic Drugs,” have particularly examined the role of early, sensitive markers of cardiac dysfunction, in order to predict this form of cardiomyopathy before left ventricular ejection fraction is reduced. It seems actually that this is increasingly important issue, along with the evaluation of novel therapeutic and cardioprotective strategies, to protect cardiooncologic patients from the development of congestive heart failure. As reported by A. Adegunsoye et al., there are also a consistent number of patients who die due to right heart failure and pulmonary hypertension secondary to fibrotic lung diseases. Significant factors which appear to play a role in the mechanism of progression of right heart dysfunction include chronic hypoxia, defective calcium handling, hyperaldosteronism, pulmonary vascular alterations, cyclic strain of pressure and volume changes, elevation of circulating TGF-*β*, and elevated systemic NO levels. The authors have reported an exhaustive review of novel therapeutic strategies for reducing right heart failure associated mortality in fibrotic lung diseases, because only “an early, effective and individualized therapy may prevent overt right heart failure in fibrotic lung disease leading to improved outcomes and quality of life.”

Another major focus of studies on acute heart failure is related to the evaluation of new surgical alternatives to transplantation or new systems or new materials available for future cardiac assist devices. In this special issue the review of J. Anand et al. explores the evolution of mechanical circulatory support and its potential for providing long-term therapy, which may address the limitations of cardiac transplantation. The innovation progresses have led to a solution of current challenges involving device complications. Moreover outcomes continue to improve and further data from both small and large registries help to advance evidence-based practices: thus patients in the most advanced stages of heart failure appear to have more hope than ever before. On the other hand the high costs, expanding indications, and rapidly increasing number of devices implanted will ultimately require important decisions to be made on the part of society, clinicians, and administrative agencies in order to establish the potential amount of economic resources to spend on this expensive, yet effective, therapy. Patient selection will remain paramount, although a very large population of patients will have the potential to benefit.

In particular, K. Unthan et al. reported a study on the design and evaluation of a fully implantable control unit for blood pumps. It is well known that pneumatic devices sufficiently supply the patients with blood flow, although the patient's quality of life is limited by the percutaneous pressure lines and the size of the external control unit. General requirements for any implantable control unit are defined from a technical and medical point of view: need for a Transcutaneous Energy Transmission, autonomous operation, safety, geometry, and efficiency. The authors described the development of the control unit of the ReinHeart, a fully implantable Total Artificial Heart that, in validation tests, is demonstrated to be a stable operation with a promising good efficiency. Finally P. Morillas-Sendín et al. assessed the effect of sevoflurane and propofol on organ blood flow in a porcine model with a left ventricular assist device. The authors demonstrated that, compared with propofol, sevoflurane increases blood flow in the brain, liver, and heart after implantation of a left ventricular assist device under conditions of partial support, giving interesting indications for the intensive pharmacological care of these high-risk patients.

The modern approach to the diagnosis and treatment of heart failure is multidisciplinary and should be based on a close collaboration among researchers, clinicians, and cardiac surgeons particularly given that mandatory multiorgan attention is required in these high-risk patients.

Future therapies for heart failure could include ventricular assist devices implantation or ventricular restoration techniques with the aim to obtain a reverse, positive remodeling in the unloaded heart. With an expanding “toolbox” of comprehensive basic, medical, surgical, and technological approaches, it is expected that these novel findings will soon be translated to the clinical practice. In fact, new therapeutic strategies are needed by the millions of patients suffering from heart failure. We hope that this special issue will help readers become familiarized with recent progress regarding the diagnosis and treatment of heart failure.

